# A Novel Type of Aspiration Presenting With Acute Respiratory Failure Caused by Upper Airway Obstruction Due to Diaper Pica

**DOI:** 10.7759/cureus.16375

**Published:** 2021-07-13

**Authors:** Noboru Hamada, Suzuka Tanaka

**Affiliations:** 1 Respiratory Medicine, Okayama City Hospital, Okayama, JPN; 2 Respiratory Medicine, National Hospital Organization Okayama Medical Center, Okayama, JPN

**Keywords:** diaper pica, dementia, direct bronchoscopic suction, high molecular weight polymers, acute respiratory failure

## Abstract

We herein report an extremely rare case of acute respiratory failure due to pica of the high-molecular-weight polymers in a diaper (HMWPD).

An 81-year-old woman was admitted to our hospital because of dyspnea. Several hours earlier, the facility staff saw her tearing the diaper and eating it. And several hours later, the staff found her complaining of dyspnea. Her chest computed tomography revealed airway foreign bodies. Immediately, flexible bronchoscopy was performed. The trachea and the bilateral bronchi were filled with the HMWPD. We tried to grasp the HMWPD with various common instruments, but the HMWPD could not be grasped. Finally, we performed direct bronchoscopic suction. All the HMWPD were removed successfully. The HMWPD have hygroscopic property resulting in volume expansion and the fatal consequence of upper airway obstruction.

Physicians working in an emergency department should be aware of this acute respiratory failure due to diaper pica and its effective treatment.

## Introduction

Foreign body aspiration has a bimodal distribution in children and elderly patients. In recent years, the number of elderly patients with foreign body aspiration has increased. On the other hand, pica is eating things that are not food, and an extremely dangerous form of self-injurious behavior exhibited by people with developmental disabilities and dementia patients [1]. Moreover, cases of life-threatening gastrointestinal obstruction due to diaper pica are sometimes reported [2]. But to the best of our knowledge, our case is an extremely rare case of an elderly dementia patient who had acute respiratory failure due to pica of the high-molecular-weight polymers in a diaper (HMWPD).

We herein report why diaper pica causes acute respiratory failure and the effectiveness of direct bronchoscopic suction in the removal of the HMWPD in the airway.

## Case presentation

**Figure 1:**
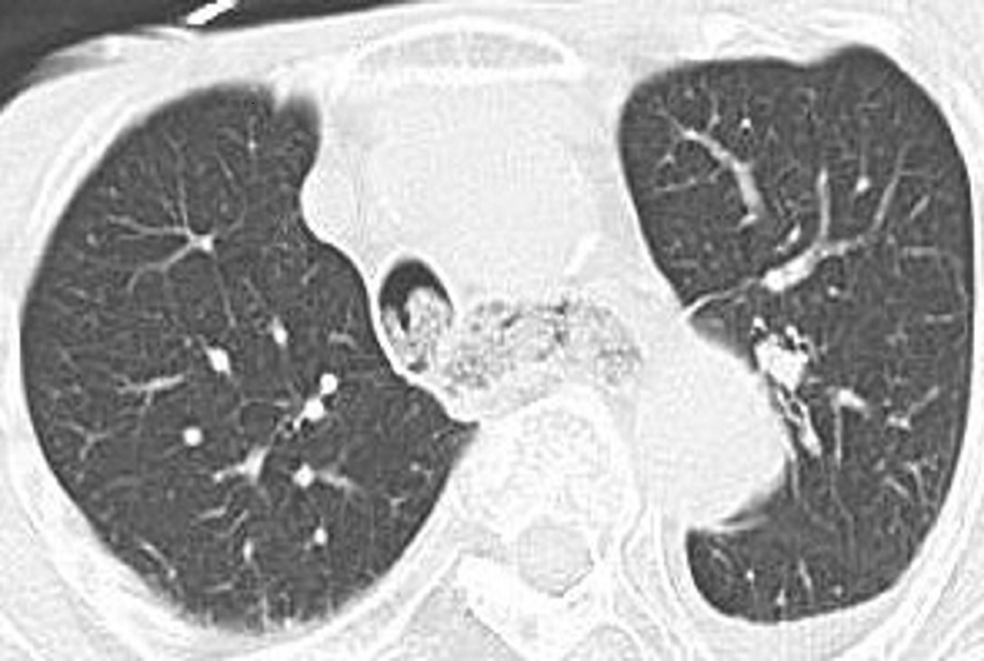
Foreign bodies filled in the trachea and esophagus on chest computed tomography

An 81-year-old woman was urgently admitted to the emergency department (ED) of our hospital with dyspnea. The patient had no psychiatric medical history but had severe dementia with a score of 3 on the clinical dementia rating. The patient also had several histories of diaper pica. A few hours ago, the facility staff confirmed that the patient was tearing the diaper and eating it, but several hours later, the staff found the patient complaining of dyspnea. On admission, physical examination revealed diminished respiratory sound. But we heard a faint stridor during inspiration. Cyanosis appeared on her lips. Her vital signs included a blood pressure of 88/54 mmHg, pulse rate of 112 per minute, respiratory rate of 18 per minute, and percutaneous oxygen saturation of 74% on room air. The arterial blood gas analysis revealed severe acidosis with pH 6.39, PaO_2_ 45 mmHg, and PaCO_2_ 150 mmHg. Laboratory data were normal with a hemoglobin level of 14.5 g/dL, C-reactive protein level of 0.03 mg/dL, while white blood cells were elevated to 16,700 µL with 73% neutrophils. The oral cavity and pharyngolarynx were filled with the HMWPD and the torn nonwoven fabric. Although these diaper materials in the oral cavity and the pharyngolarynx were removed using the grasping forceps under the laryngoscope, respiratory failure did not improve. Therefore, it was assumed that these diaper materials had fallen from the pharyngolarynx to the trachea. Endotracheal intubation was performed immediately. Her chest roentgenogram was normal, but her chest computed tomography revealed foreign bodies in the trachea and esophagus (Figure 1).

**Figure 2:**
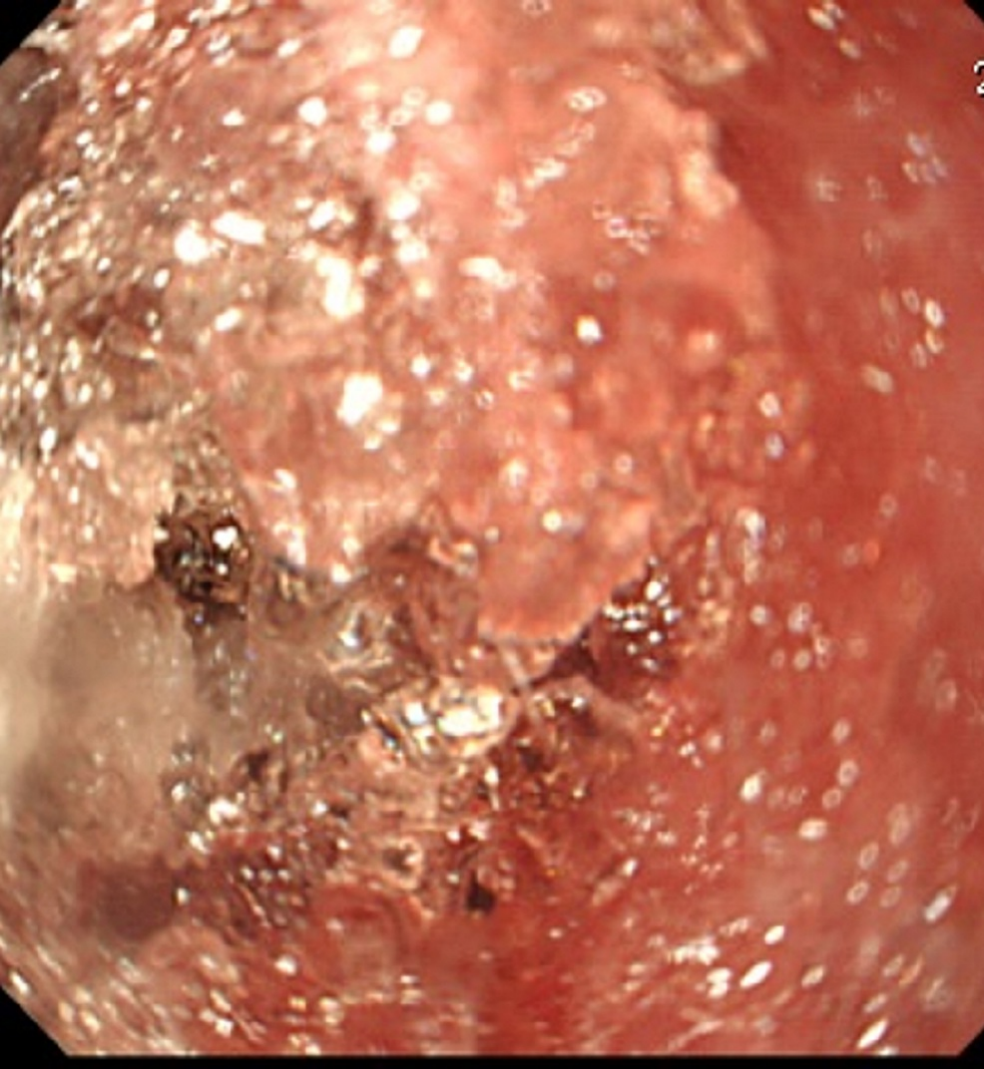
High-molecular-weight polymers in diaper filled in the trachea on bronchoscopy

Flexible bronchoscopy was performed, and the HMWPD were visualized from the trachea to the main bronchi bilaterally (Figure 2).

We tried to grasp the HMWPD in a lump with grasping and basket forceps in order, but the HMWPD were gelled and could not be grasped (as they were broken into pieces). Finally, we performed direct bronchoscopic suction. As a result, all the HMWPD were removed, and the bronchoscopic suction port was not blocked by the HMWPD. During flexible bronchoscopy, percutaneous oxygen saturation was maintained at 96% under the oxygen control of endotracheal intubation. The esophagus and stomach were also filled with the HMWPD and the torn nonwoven fabric. Subsequently, the torn nonwoven fabric was removed with grasping forceps and the HMWPD were also removed by direct upper endoscopic suction in the same procedure. After bronchoscopy, the arterial blood gas analysis revealed pH 7.39, PaO_2_ 111 mmHg, and PaCO_2_ 49 mmHg under 2 L oxygen inhalation. Since her respiratory condition was stable after the treatment, the patient was extubated. We could not do the swallowing function evaluation studies because we could not get her help. Then, her vital signs included a blood pressure of 110/74 mmHg, and percutaneous oxygen saturation of 96% on room air. She left hospital the next day.

## Discussion

We have found two clinical issues. First, the HMWPD can be an airway foreign body causing acute respiratory failure. Second, direct bronchoscopic suction is effective in removing the HMWPD. 

The first is that a case of acute respiratory failure due to diaper pica was extremely rare in our investigation. The HMWPD increase 100 times in volume when they contain water. And it is said that the expanded hygroscopic HMWPD are difficult to recover to original form even under external pressure. Therefore, the volume of the HMWPD entering the trachea increases gradually with moisture in the airway. It leads to airway obstruction and respiratory failure several hours after aspiration.

It also leads to an extremely dangerous clinical course that is not noticed by anyone. On the other hand, Don et al. and Fang et al. reported 200 and 96 Chinese adults with airway foreign bodies, respectively [3,4]. In those reports, there are two kinds of types of airway foreign bodies. One is an acute airway foreign body presenting with acute respiratory failure immediately after aspiration. The other is a chronic airway foreign body detected incidentally during bronchoscopy for further examination of recurrent aspiration pneumonia or atelectasis [4]. Our case is an acute airway foreign body. However, our case is a new type of acute airway foreign body occurring several hours after aspiration, rather than that occurring immediately after aspiration. And another kind of type of airway foreign body is that organic substance type and inorganic substance type. Diaper is classified as an inorganic one. However, teeth are the most common inorganic airway foreign bodies. This is because most patients can easily find their lost original or prosthetic teeth by chest roentgenogram. Diaper pica was not reported in those reviews. It may be because it is hard to detect by chest roentgenogram. We report a unique clinical course of severe respiratory failure that occurred several hours after aspiration due to the diaper material, which has not been mentioned anywhere.

The second is that direct bronchoscopic suction was effective in removing the HMWPD. Don et al. reported that flexible bronchoscopy was effective in removing airway foreign bodies. The review reported a success rate of about 96.5% [3]. Commonly used instruments include grasping forceps for relatively hard objects, basket forceps for soft objects, Fogarty catheters for fragile objects [5], and snare loops for relatively large objects [6]. At first, we used these common instruments, but the HMWPD could not be removed. All the HMWPD could be removed by direct bronchoscopic suction without occluding the bronchoscopic suction port. Possible reasons for the successful removal of the HMWPD are as follows: the interparticle binding force of the HMWPD may be comparatively weak, and the particles of the HMWPD are easily separated by the bronchoscopic suction force. This can be predicted from the fact that the HMWPD were broken into pieces when they were held by grasping forceps. This direct bronchoscopic suction method is simple, but it is hard to come up with this method right away working in an ED. However, this approach has not yet been fully proven to be truly effective, because such cases have rarely been reported. We would like to prove the effectiveness of the treatment by experiencing more cases in the future.

## Conclusions

We encountered an extremely rare case of acute respiratory failure due to diaper pica. In this case, all the HMWPD were successfully removed by direct bronchoscopic suction. It is expected that such cases will increase in an aging society, and physicians working in an ED should recognize this dangerous clinical course and its effective treatment.
